# Inhibition of IκBα phosphorylation potentiates regulated cell death induced by azidothymidine in HTLV-1 infected cells

**DOI:** 10.1038/s41420-020-0243-x

**Published:** 2020-02-18

**Authors:** Claudia Matteucci, Francesca Marino-Merlo, Antonella Minutolo, Emanuela Balestrieri, Elena Valletta, Beatrice Macchi, Antonio Mastino, Sandro Grelli

**Affiliations:** 1 Department of Experimental Medicine, University of Rome “Tor Vergata”, Rome, Italy; 2 IRCCS Centro Neurolesi Bonino-Pulejo, Messina, Italy; 3 Department of Chemical Science and Technologies, University of Rome “Tor Vergata”, Rome, Italy; 4 Department of Chemical, Biological, Pharmaceutical, and Environmental Sciences, University of Messina, Messina, Italy; 5 The Institute of Translational Pharmacology, CNR, Rome, Italy

**Keywords:** Preclinical research, Cancer therapy

## Abstract

Adult T cell leukemia/lymphoma (ATL) can be susceptible, at least transiently, to treatments with azidothymidine (AZT) plus IFNα and/or arsenic trioxide. However, the real role of AZT in this effect is still unclear. In fact, while reverse transcriptase (RT) inhibition could explain reduction of clonal expansion and of renewal of HTLV-1 infected cells during ATL progression, this effect alone seems insufficient to justify the evident and prompt decrease of the pro-viral load in treated patients. We have previously demonstrated that AZT is endowed with an intrinsic pro-apoptotic potential towards both peripheral blood mononuclear cells from healthy donors or some tumor cell lines, but this cytotoxic potential cannot be fully achieved unless IκBα phosphorylation is inhibited. Since the constitutive activation of NF-kappa B (NF-κB) appears a common biological basis of HTLV-1-infected cells, a pharmacological inhibition of IκBα phosphorylation seems a potential strategy for treating and preventing HTLV-1 related pathologies. In this study, we have demonstrated that a combination treatment with the IκBα phosphorylation inhibitor Bay 11-7085 and AZT induced increased levels of regulated cell death (RCD) by apoptosis compared to the single treatments in HTLV-1 infected cells of different origin. Importantly, levels of RCD were considerably higher in infected cells in comparison with the uninfected ones. Inhibition of NF-κB activation following the combined treatment was confirmed by analysis of both gel-shift and functional activity of the NF-κB complex proteins, p65/p52. Moreover, a transcriptional analysis revealed that the addition of Bay 11-7085 to AZT treatment in HTLV-1-infected cells modified their transcriptional profile, by inducing the upregulation of some pro-apoptotic genes together with the downregulation of some anti-apoptotic genes. Our data suggest that addition of adequate concentrations of IκBα phosphorylation inhibitor to therapeutic regimens including AZT could be a promising strategy in ATL.

## Introduction

Human T-lymphotropic virus type 1 (HTLV-1) is the etiological agent of both hematologic and neurologic diseases. The most severe hematologic disorder caused by HTLV-1 is adult T-cell leukemia/lymphoma (ATL), a malignancy of CD4+ T-cells^1^. Regarding to neurologic disorders, HTLV-1 infected individuals could develop HTLV-1-associated myelopathy/tropical spastic paraparesis (HAM/TSP) or other inflammatory based diseases^2–4^. HTLV-1 is diffused in endemic area in Central and Southern America, Central and Southern Africa, Middle Est, Japan, or in specific local communities, as recently found for some Aboriginal Australians groups^5,6^. Nevertheless, due to increasing migration flows, geographical distribution of the virus has become ever greater and less discernible. About 5–10 millions of people are estimated to be infected worldwide. However, only a total of up to 10% develop HTLV-1 associated diseases after a period of long latency. In fact, HTLV-1 persistence is characterized by profound interactions of virus regulatory proteins with host cell factors driving cell death/proliferation^7,8^, and by activation of the immune system^9^. Efficacious therapeutic approaches towards all forms of HTLV-1 related diseases and/or prophylactic or therapeutic vaccines are not currently available. Monotherapy with reverse transcriptase (RT) inhibitors, already used towards HIV, did not substantially change the course of HTLV-1 associated diseases^10,11^. Therefore, the implementation of health interventions to control HTLV-1 infection is a great challenge for the future^12^. In particular, ATL is resistant to classical chemotherapy and the only effective therapy at the moment, although not curative, is a regimen including combined treatment of azidothymidine (AZT) plus interferon alpha (IFNα)^13,14^ and or arsenic trioxide^15–18^. Nevertheless, mechanisms underlying the partial efficacy of this combination, await further clarifications^19,20^. Our previous studies have demonstrated that AZT and other nucleoside compounds act as potent RT inhibitors towards HTLV-1 RT activity in vitro^21–23^. Obviously, however, RT inhibition cannot affect already infected ATL clones. Interestingly, recent results suggest that a continuous reinfection of HTLV-1 clones occurs during leukemogenesis in ATL^24,25^. Inhibition of horizontal virus transmission by AZT could be hindered, anyhow, by continuous growth of ATL cells favored by mechanisms counteracting regulated cell death (RCD). The latter include activation of the transcription factor nuclear factor-κB (NF-κB)^26^, tightly regulated by the HTLV-1 proteins Tax and basic leucine zipper (bZIP) factor (HBZ)^27–29^.

We have observed that, in addition to its RT inhibitory activity, AZT has the capability to activate conflicting apoptosis-related signals. However, only when AZT was combined with pharmacological inhibition of IκBα phosphorylation, cells were actually pushed towards apoptosis^30–32^. The aim of the present study was to investigate whether the pharmacological inhibition of IκBα phosphorylation could potentiate a possible pro-apoptotic effect exerted by AZT towards HTLV-1 infected cells. To this purpose, we analyzed the susceptibility to apoptotic RCD of HTLV-1-infected cell lines following a combination treatment with AZT and Bay 11-7085.

## Results

### Pro-apoptotic effects of AZT towards normal and HTLV-1-transformed cells

**Fig. 1:**
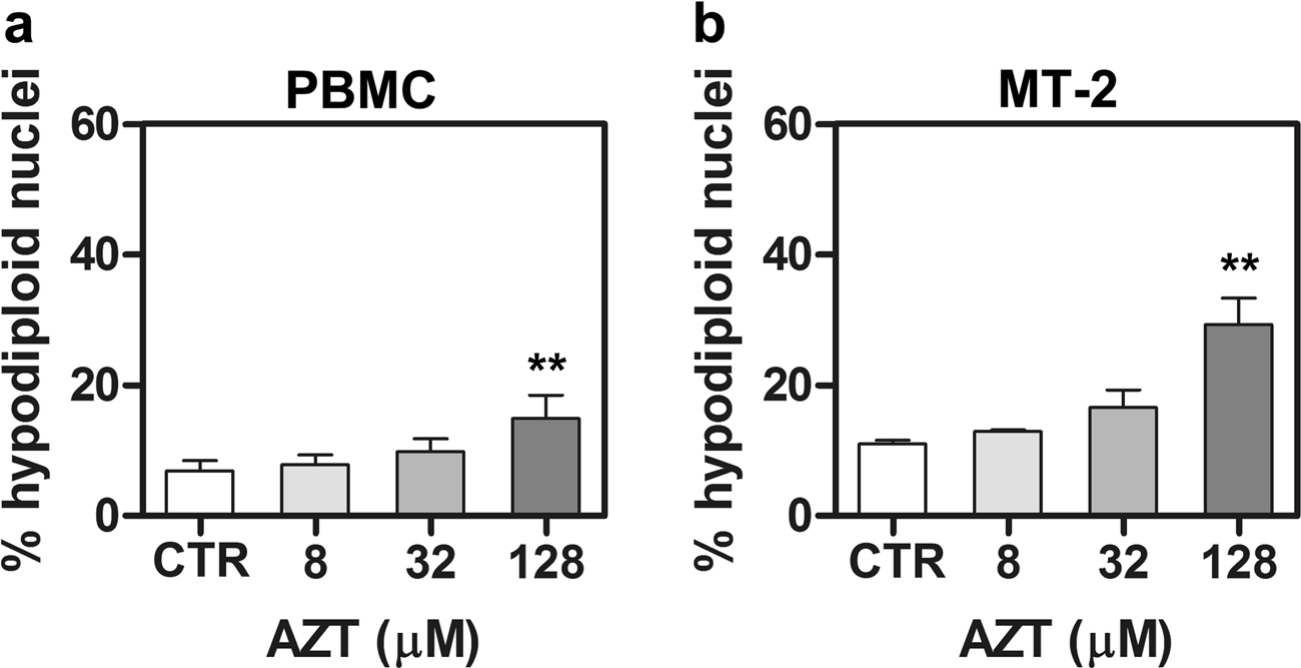
Apoptotic RCD induced by AZT towards PBMC from healthy donors and MT-2 cells. Percentages of hypodiploid nuclei, detected by flow cytometry, in AZT treated PBMC (**a**) and MT-2 cells (**b**). The cells were treated with medium as a vehicle (CTR) or treated with 8, 32, and 128 µM AZT for 72 h. The results are expressed as mean values ± S.D. obtained from three independent experiments. Asterisks indicate significant (*p < 0.05) and highly significant (**p < 0.001) differences between treated and control cells.

In order to evaluate the pro-apoptotic potential of AZT towards normal and HTLV-1-transformed cells, PBMC from healthy donors and cells from the chronically HTLV-1-infected MT-2 cell line, were treated with the drug at concentrations up to 128 µM. Percentages of hypodiploid nuclei were then evaluated at 72 h. The timing of these experiments was based on previous experience indicating that a time of 24 h after treatment was too early to observe reasonable levels of apoptosis, both in PBMC and in MT-2 cells. PBMC, as well as MT-2 cells, showed a low response to apoptosis induction when exposed to AZT in vitro. In PBMC, an evident increase in percentage of hypodiploid nuclei, in comparison with control cells, was observed only at the higher concentration of 128 µM (Fig. 1a). In MT-2 cells, an even more noticeable increase of apoptosis occurred at 128 µM in comparison with control cells (Fig. 1b). This effect was highly specific without any sign of induction of necrotic cell death. Taking into account that levels of apoptosis induced by AZT towards PBMC from healthy donor at the same concentration and time, even if recognizable, were less evident, the concentration of 128 µM AZT was considered for a possible combination treatment.

### Effects of pharmacological inhibition of IκBα phosphorylation on apoptotic regulated cell death in normal and HTLV-1-transformed cells

**Fig. 2:**
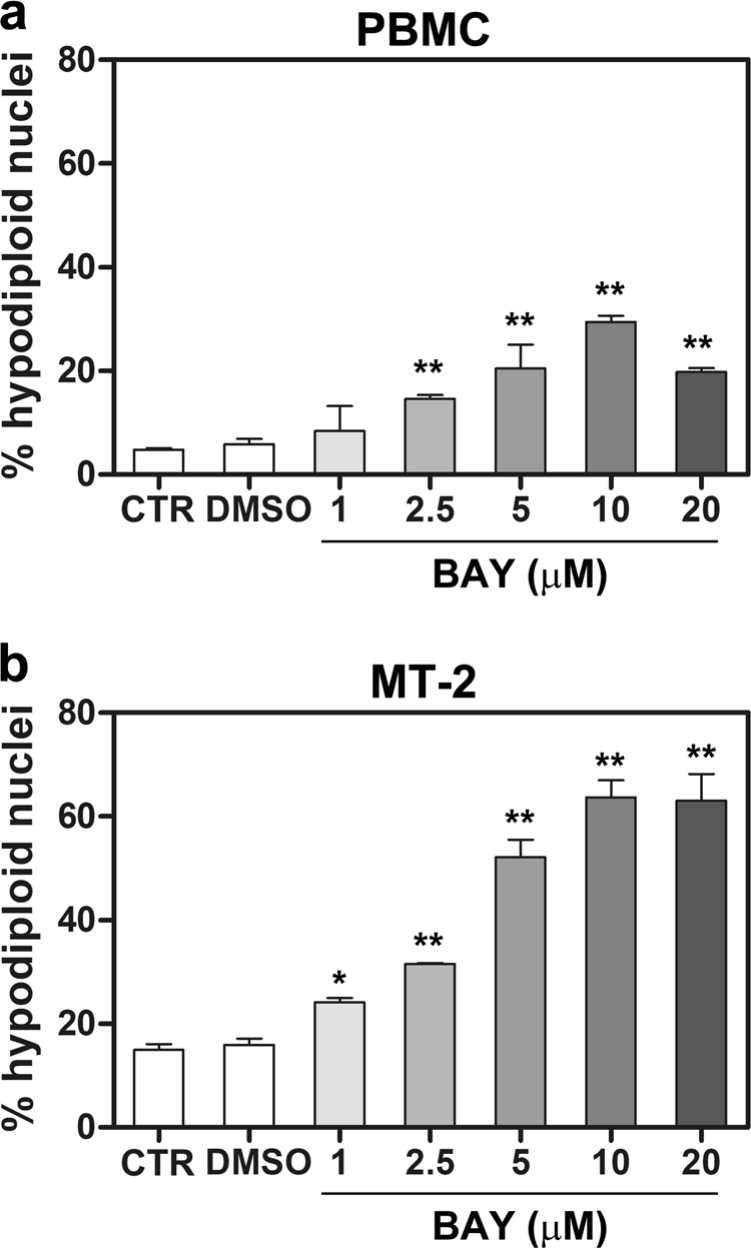
Apoptotic RCD induced by pharmacological inhibition of IκBα phosphorylation towards PBMC from healthy donors and MT-2 cells. Percentages of hypodiploid nuclei, detected by flow cytometry analysis, were assessed in PBMC (**a**) and MT-2 (**b**) cells either treated with medium as a vehicle (CTR) or treated with medium plus DMSO at the higher concentration utilized for diluting Bay 11-7085 (DMSO), or treated with 1, 2.5, 5, 10, and 20 μM Bay 11-7085, for 24 h. The results are expressed as mean values ± S.D. obtained from three independent experiments. Asterisks indicate significant (*p < 0.05) and highly significant (**p < 0.001) differences between treated and CTR cells.

To possibly potentiate the pro-apoptotic activity of AZT towards HTLV-1 transformed cells by means of the preventive inhibition of NF-κB activation, we performed dose–effect experiments to assess the ability of Bay11-7085, a pharmacological inhibitor of IκBα phosphorylation, to induce apoptosis at 24 h. In PBMC, Bay 11-7085 induced an evident, dose-dependent increase of hypodiploid nuclei at all the concentrations tested, except at 1 µM, in comparison with vehicle (Fig. 2a). Regarding to MT-2 cells (Fig. 2b), Bay 11-7085 even at the lowest concentrations of 1 and 2.5 µM induced noticeable hypodiploid nuclei percentages. Levels of apoptosis dramatically increased at higher concentrations, reaching a plateau of about 63% at 10 μM. Given the necessity to limit the risk of toxicity by Bay 11-7085, the suboptimal concentration of 1 µM was chosen for the following combination experiments with AZT.

### Susceptibility of HTLV-1-transformed cells to apoptotic regulated cell death induced by a combination treatment with AZT and an inhibitor of IκBα phosphorylation

**Fig. 3:**
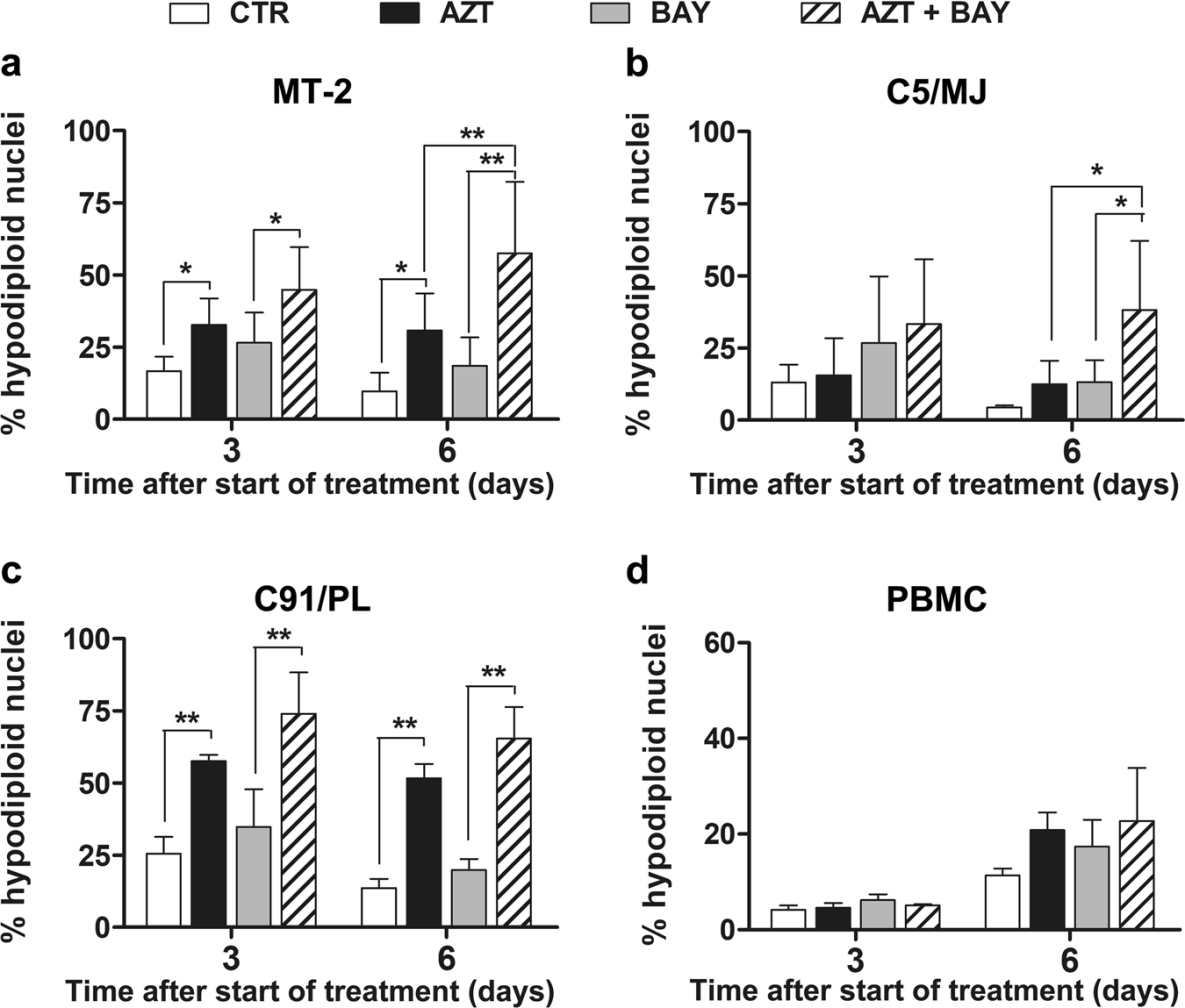
Apoptotic RCD induced by a combination treatment with AZT and an inhibitor of IκBα phosphorylation towards HTLV-1 chronically infected cell lines. Percentages of hypodiploid nuclei, detected by flow cytometry, in MT-2 (**a**), C5/MJ (**b**), and C91/PL (**c**) cells, or PBMC from healthy donors (**d**), after treatment with vehicle (CTR), with 128 µM AZT (AZT), with 1 µM Bay 11-7085 (BAY), or pre-treatment with 1 µM Bay 11-7085 for 2 h and subsequent treatment with 128 µM AZT (AZT+BAY) for 72 h, i.e. after a total of 3 days in culture (day 3) or following a second retreatment with the same protocol for another 72 h, i.e. after a total of 6 days in culture (day 6). The results are expressed as mean values ± S.D. obtained from three independent experiments. Asterisks indicate significant (*p < 0.05) and highly significant (**p < 0.001) differences between groups (connection bars).

We then wanted to investigate the effect of a combined treatment consisting of AZT preceded by inhibition of IκBα phosphorylation towards cells transformed by HTLV-1. To this purpose, in addition to MT-2 cells, two others chronically infected cell lines, amply utilized for studies on HTLV-1, designated as C5/MJ and C91/PL, were utilized. All the three cell lines were pre-treated with Bay 11-7085 at 1 µM for 2 h and subsequently treated with 128 µM AZT. The percentage of hypodiploid nuclei was then assessed after a further incubation of 72 h, i.e. after 3 days in culture, as well as following a retreatment of the cells with the same protocol for another 72 h, i.e. after a total of 6 days in culture. An increase of apoptosis levels in MT-2 cells following the combination treatment, compared to single treatments, was already evident after 3 days (Fig. 3a). After 6 days in culture and two cycles of combination treatment, levels of apoptosis of MT-2 cells were much more dramatically enhanced with respect to those induced by single treatments (Fig. 3a). The C5/MJ cell line was found to be less susceptible than MT-2 cells to AZT alone both after 3 days and after 6 days treatment (Fig. 3b). At 3 days, the preventive addition of Bay 11-7085 to AZT treatment induced indeed an increased level of apoptosis with respect to single treatments, but differences were not statistically significant, probably due to high variability among the independent experiments. Conversely, after 6 days in culture and the two-cycle combination treatment, apoptosis induced by AZT in C5/MJ cells was noticeably increased by preventive Bay 11-7085 addition with respect to AZT alone. Regarding to the C91/PL cell line, cells showed to be very sensitive to apoptosis induced by AZT alone, both after 3 and 6 days in culture. Combined treatment with AZT and Bay 11-7085, resulted indeed in a higher apoptotic response at 3 as well as at 6 days (Fig. 3c). However, due to elevated response to AZT alone, differences with respect to AZT alone were not statistically significant (Fig. 3c). To verify whether the combined treatment could be toxic for uninfected normal cells, PBMC from healthy individuals were subjected to the same protocol utilized for HTLV-1 transformed cells. The results showed that the AZT treatment either in the absence or in the presence of 1 µM Bay 11-7085 did not induce evident levels of hypodiploid nuclei at 3 days in culture (Fig. 3d). After 6 days in culture, apoptosis level following the combined treatment, although increased with respect to the previous timing, was still relatively low, and no statistically significant difference with respect to control cells was assessed.

To confirm that increased levels of hypodiploid nuclei following combination treatment with AZT and Bay 11-7085 in HTLV-1 infected cells were actually related to the apoptotic form of RCD, further experiments on MT-2 cells were carried out using the Annexin-V/PI staining, as detected by flow cytometry, and cleavage of caspase 3 and PARP-1, as detected by Western blot analysis, as additional markers of apoptotic cell death. Results, reported in Supplementary Information 2, clearly confirmed that the combination treatment with AZT and Bay 11-7085 induced higher levels of cell death in HTLV-1 infected cells with respect to single treatments and that cells underwent cell death with the classical feature of apoptosis. Therefore, altogether these results showed a consistent, although variable, response of HTLV-1 transformed cells to apoptotic RCD induced by a combination treatment with AZT preceded by an inhibitor of IκBα phosphorylation, while the same combination treatment did not substantially change levels of apoptosis in PBMC from healthy individuals.

### Effects of the combination treatment with AZT and an inhibitor of IκBα phosphorylation towards cells infected in vitro by HTLV-1, but not yet transformed

**Fig. 4:**
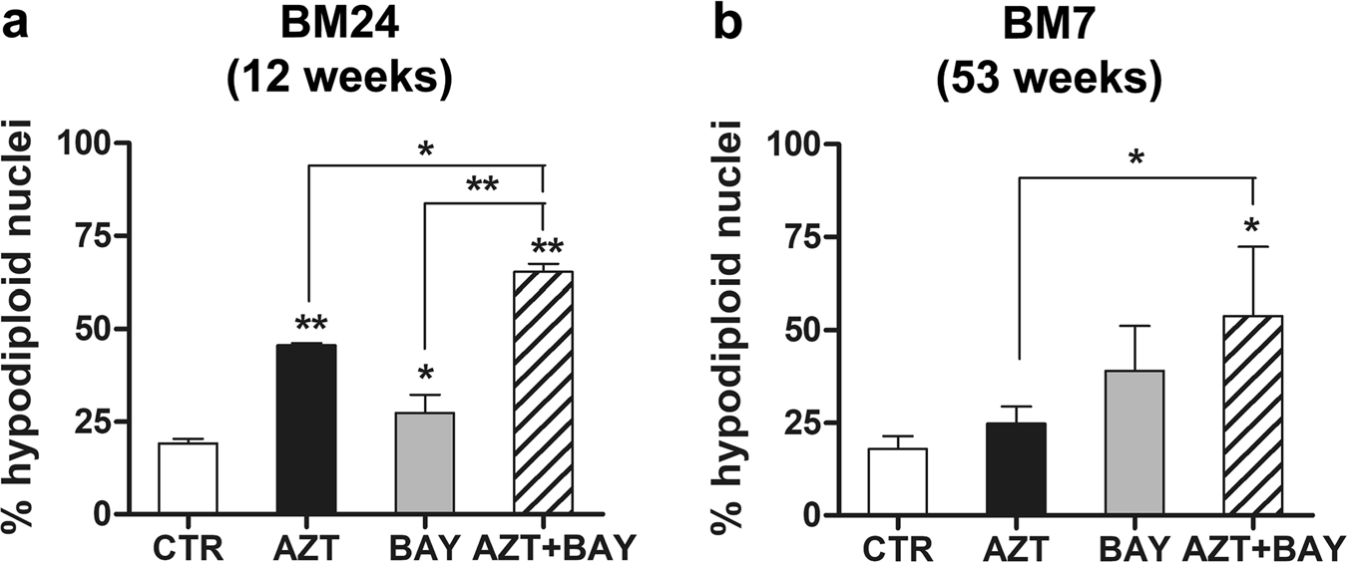
Apoptotic RCD induced by a combination treatment with AZT and an inhibitor of IκBα phosphorylation towards not-yet-transformed, in vitro HTLV-1 infected cells. Percentages of hypodiploid nuclei were assessed in IL-2 dependent, in vitro HTLV-1 infected BM24 (**a**) and BM7 (**b**) cell cultures, at 12 and 52 weeks in culture, respectively, from infection, following treatment with vehicle (CTR), with 128 µM AZT (AZT), with 1 µM Bay 11-7085 (BAY), or pre-treatment with 1 µM Bay 11-7085 for 2 h and subsequent treatment with 128 µM AZT (AZT+BAY) for 72 h. The results are expressed as mean values ± S.D. obtained from three independent experiments. Asterisks indicate significant (*p < 0.05) and highly significant (**p < 0.001) differences referred to CTR cells (no connection bar) or between groups (connection bars).

To get information on the effects of the combination treatment with AZT and the inhibitor of IκBα phosphorylation on cells infected by HTLV-1 during the phase that precede the eventual transforming event, we used IL-2-dependent cell cultures infected in vitro with HTLV-1 in our laboratory, at different times a.i.. In particular, BM24, at 12 weeks a.i., and BM7, at 52 weeks a.i., were assayed at 72 h after treatment. Regarding to BM24, preventive inhibition of NF-κB activation rendered these cells high susceptible to apoptosis induced by AZT in comparison with AZT alone (Fig. 4a). Similarly, also BM7 cells were highly susceptible to the induction of apoptotic RCD by the combination treatment (Fig. 4b). However, presumably due to high inter-experimental variability and to high level of apoptosis induced Bay 11-7085 alone, no statistical difference was observed between BM7 cells treated with the inhibitor of IκBα phosphorylation alone and the combination treatment. Thus, also in cells infected in vitro by HTLV-1, but not-yet-transformed, the apoptotic response to the combination treatment was consistently higher in comparison with that obtained by AZT alone.

### Effects of the combination treatment with AZT and an inhibitor of IκBα phosphorylation on NF-κB activation

**Fig. 5:**
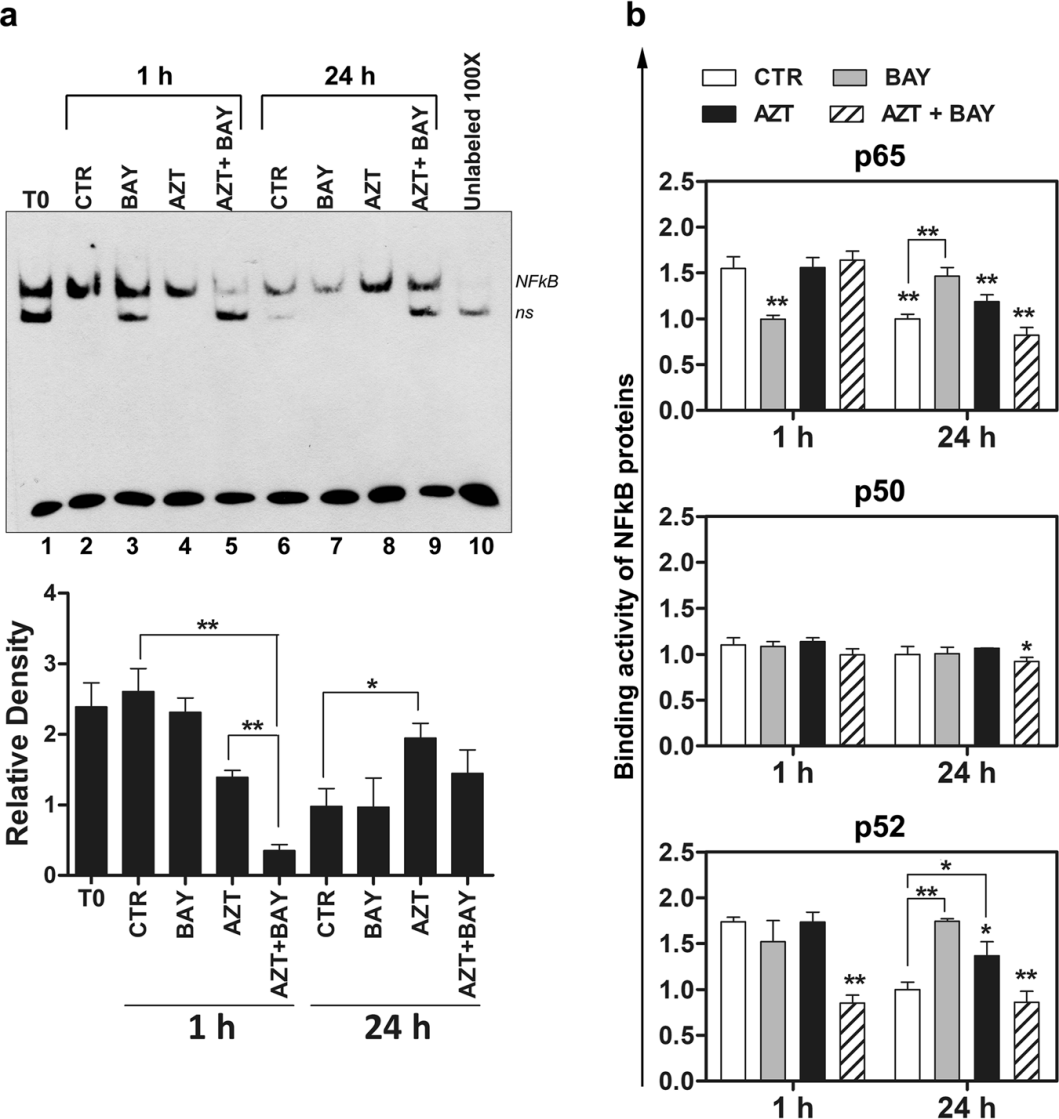
Detection of NF-κB activation in MT-2 cells treated with AZT and an inhibitor of IκBα phosphorylation. MT-2 cells were either treated with vehicle (CTR) or treated with 1 µM Bay 11-7085 alone (BAY), 128 µM AZT alone (AZT), or with both (AZT+BAY), and then assayed at 1 and 24 h after the last treatment for NF-κB DNA-binding activity using non-radioactive electro-mobility shift assay (EMSA) (**a**), or detection of phosphorylated p65, p50 and p52 binding by an enzyme-linked immunosorbent assay (ELISA) (**b**). **a** A representative gel (upper side) and results of densitometry analysis (lower side) expressed as mean values ± S.D. obtained from three gels generated in independent experiments. T0 sample refers to cells assayed by immediately before starting any treatment. **b** The histograms represent the mean values ± S.D. from three independent experiments and are expressed as the ratio of the values obtained from samples of the experimental groups versus those obtained in samples of the CTR group at 24 h. Asterisks indicate significant (*p < 0.05) and highly significant (**p < 0.001) differences referred to the CTR group at 1 h (no connection bar) or between groups (connection bars).

To investigate whether effects exerted by the combination treatment were associated to the modulation of the NF-κB complex, MT-2 cells subjected to various treatments were assayed immediately before starting any treatment (T0), and at 1 and 24 h after the last treatment. Firstly, NF-κB activation was detected using EMSA (Fig. 5a). As assessed by the comparison of the bands of the treated samples and of the corresponding control samples, the inhibition of NF-κB complex activation remarkably occurred early after 1 h following the combination treatment. AZT treatment itself induced a slighter inhibition of NF-κB activation at the early time, but an increase of the NF-κB complex at the late time, after 24 h, suggesting that the cells were in a rebounding phase from the first AZT treatment. In fact, in this case the addition of Bay 11-7085 partly counteracted the AZT-driven rebound, so that a lower difference, with respect to control cells, was observed at 24 h in comparison with AZT alone treated cells. On the contrary, the single treatment with Bay 11-7085 was unable to induce relevant changes in comparison with control at the detection times utilized for these experiments (Fig. 5a). Notice that differences in the band feature of CTRL samples at different times of treatment indicates that basal levels of NF-κB varied depending on the growth phase in culture and strongly recommended to compare treated samples limited to corresponding CTRL sample. We then sought to achieve further details on this issue by analyzing the DNA-binding activity of the single p65, p50, and p52 phosphorylated proteins in the cellular nuclear lysates from MT-2 cells treated as for EMSA. The results, shown in Fig. 5b, demonstrated that the DNA-binding activity of phosphorylated p50 was not modulated with respect to the control in any of the experimental conditions tested, except for the combination treatment at 24 h. Conversely, the DNA-binding activity of phosphorylated p65 and p52 was remarkably modulated in the different culture conditions. Actually, Bay 11-7085 inhibited p65 binding at 1 h after treatment with respect to control. Conversely, the same single treatment increased p65 binding at 24 h in comparison with the corresponding control, while only slight differences were observed between AZT and AZT plus Bay 11-7085 treated and control samples, with cells subjected to the combination treatment showing the lower levels of p65 binding. Regarding p52, the results showed a noticeable downregulation of this activity at 1 h following the combination treatment with respect to the control, while no evident modification after single treatments. At 24 h after treatment, the DNA-binding activity of p52 was higher in comparison with corresponding control following single treatments, but not following the combination treatment. These assays were performed also in C5/MJ cells, where we obtained a trend in the effects of single and combination treatments that was similar to that observed in MT2 cells, except for the significance of some differences and for the effect of the combination treatment at 24 h, where a significant recovery in the binding activity of the p65 protein was observed (see Supplementary Information 3). In summary, these results indicate that the eventual effect of the combination treatment on the activation of the NF-κB complex should be considered the sum of the different balance of both p65 and p52 but not, or less importantly, p50 activities.

### Effects of the combination treatment with AZT and an inhibitor of IκBα phosphorylation on the expression of cellular and viral genes

**Fig. 6:**
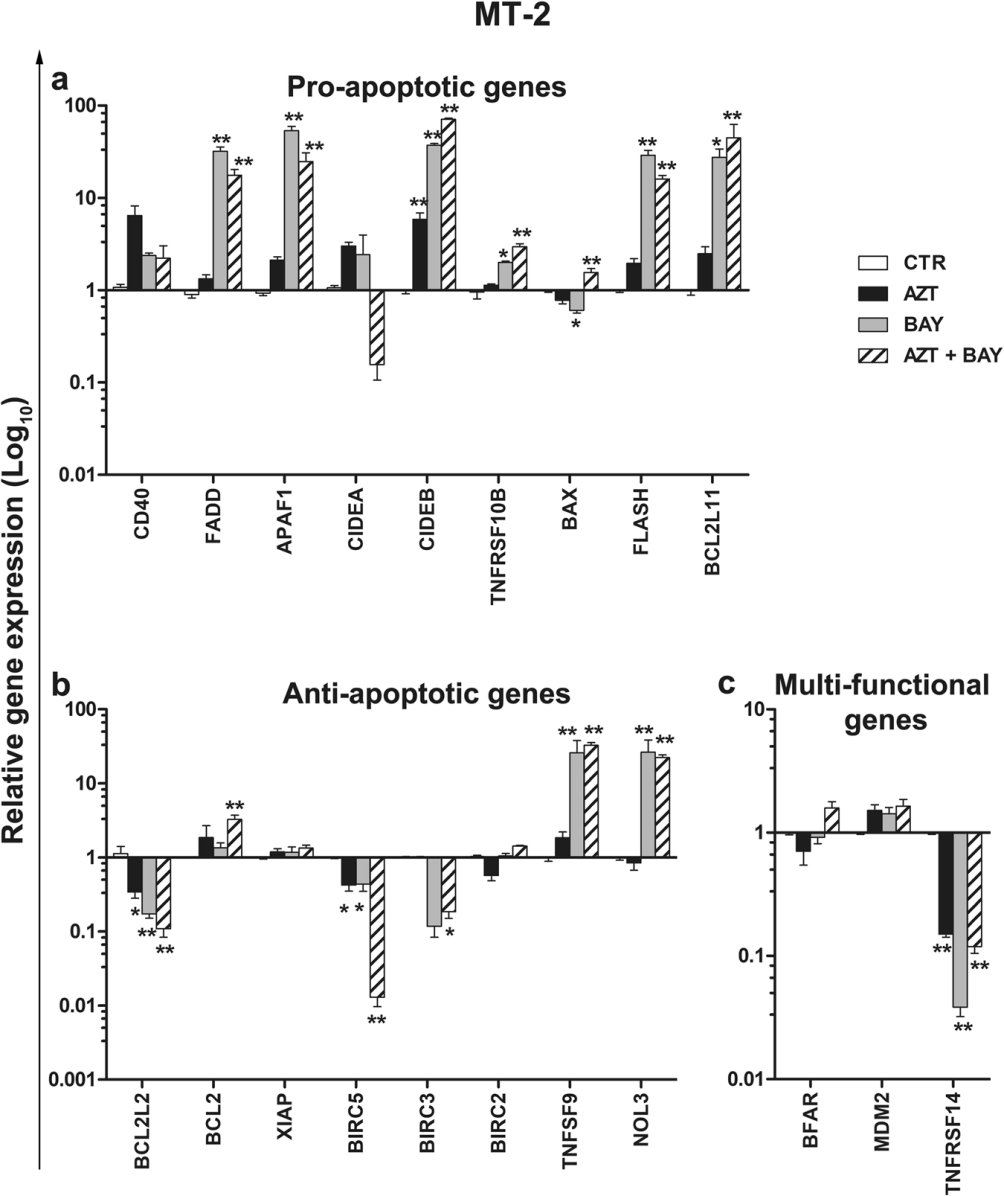
RQ-PCR analysis of apoptosis-related gene expression in MT-2 cells treated with AZT and an inhibitor of IκBα phosphorylation. MT-2 cells were either treated with vehicle (CTR) or treated with 128 µM AZT alone (AZT), with 1 µM Bay 11-7085 alone (BAY), or with both (AZT+BAY), and then assayed 24 h after the last treatment for gene-expression by real-time quantitative reverse transcription PCR (RQ-PCR). Normalization of crude values with the GUSB gene as a housekeeping gene, was performed. Relative gene expression of genes grouped as pro-apoptotic (**a**), anti-apoptotic (**b**), or multi-functional (**c**), was calculated versus time 0 control samples and is expressed as (log _10_). The histograms represent the mean values ± S.D. from three independent experiments. Asterisks indicate significant (*p < 0.05) and highly significant (**p < 0.001) differences referred to time 24 h CTR samples.

**Fig. 7:**
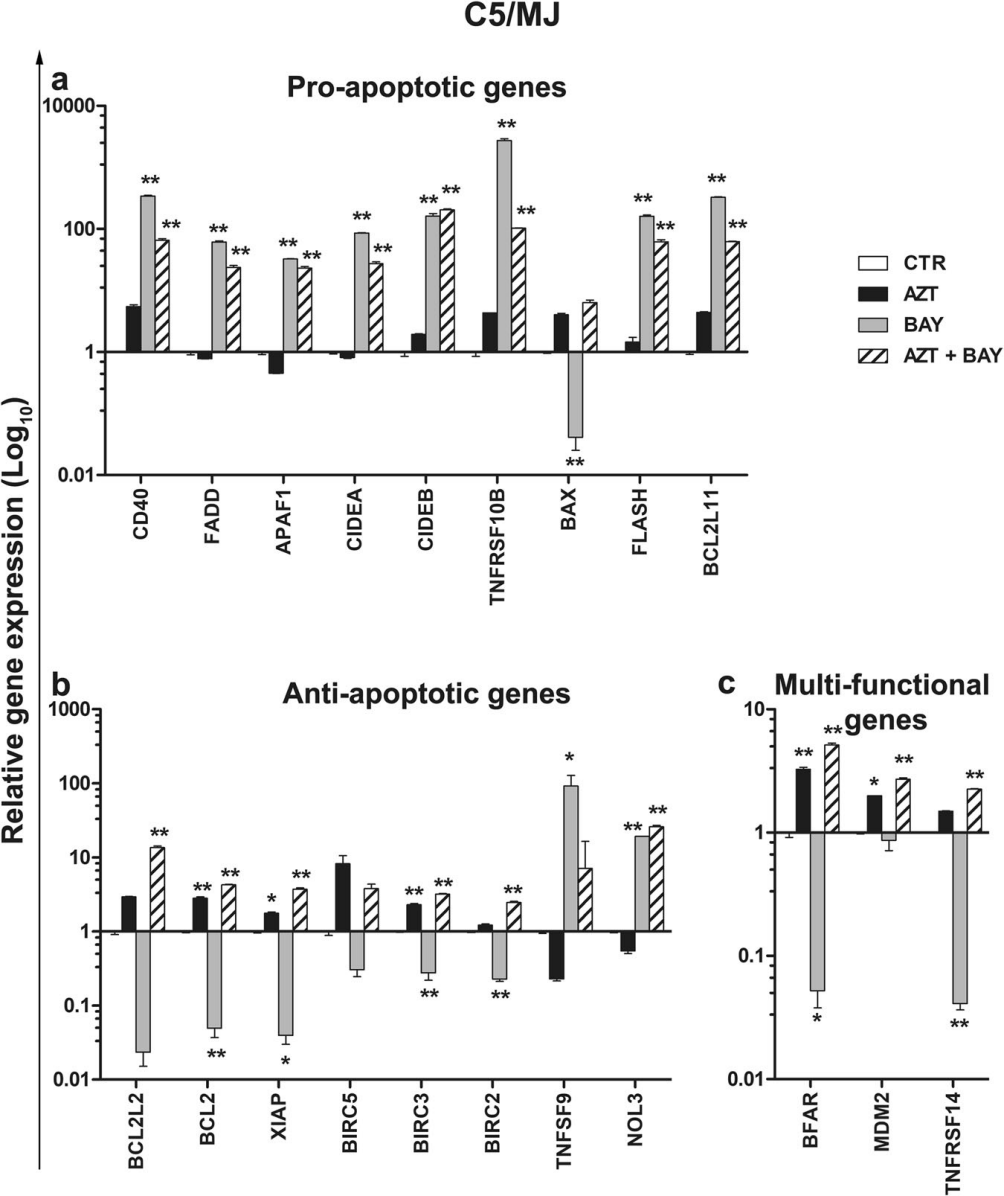
RQ-PCR analysis of apoptosis-related gene expression in C5/MJ cells treated with AZT and an inhibitor of IκBα phosphorylation. C5/MJ cells were either treated with vehicle (CTR) or treated with 128 µM AZT alone (AZT), with 1 µM Bay 11-7085 alone (BAY), or with both (AZT+BAY), and then assayed 24 h after the last treatment for gene-expression by real-time quantitative reverse transcription PCR (RQ-PCR). Normalization of crude values with the GUSB gene as a housekeeping gene, was performed. Relative gene expression of genes grouped as pro-apoptotic (**a**), anti-apoptotic (**b**), or multi-functional (**c**), was calculated versus time 0 control samples and is expressed as (log _10_). The histograms represent the mean values ± S.D. from three independent experiments. Asterisks indicate significant (*p < 0.05) and highly significant (**p < 0.001) differences referred to time 24 h CTR samples.

In order to get further information on mechanisms involved in the effects of the combination treatment with AZT plus Bay 11-7085 on RCD in HTLV-1-transformed cells, the expression of relevant cellular genes was investigated. As a preliminary experimental approach, a commercial gene array for analysing the expression profile of genes related to apoptosis and cell cycle was employed on MT-2 cells treated for 24 h. The results showed that the combination treatment induced the modulation of both pro- and anti-apoptotic genes (Supplementary Information 4). To confirm results obtained with the SuperArray analysis, a selected number of pro- and anti-apoptotic genes (underlined in Supplementary Information 4) recognized as targets of NF-κB, were chosen for quantitative analysis of gene expression by RQ-PCR in MT-2 or C5/MJ cells, at 24 h after single or combined treatments. The relative mRNA level analysis confirmed the modulation of both pro- and anti-apoptotic genes following different treatments, in both the cell lines. In MT-2 cells, a general upregulation of genes associated with cell death induction pathways in response to the treatments was observed (Fig. 6a). In particular, levels of mRNA for all genes designated as pro-apoptotic, except for BAX, were slightly higher than control samples in response to the single treatment with AZT. Conversely, the expression of the same genes was considerably upregulated, in respect to the control samples, in response to single treatment with Bay 11-7085 or to the combination treatment, except for the *CD40* and *CIDEA* genes. Specularly, some anti-apototic genes such as *BCL2L2/BCL-W*, and *BIRC5/survivin* were evidently downregulated following single treatment with AZT, but even more remarkably following the combination treatment (Fig. 6b). Also *BIRC3/c-IAP2* was downregulated, in comparison with control samples, but less extensively. Conversely, the expression of other anti-apoptotic genes, such as *TNFS9* and *NOL3*, was significantly increased in response to both Bay 11-7085 single treatment and the combination treatment, in comparison with control samples (Fig. 6b). The bifunctional regulator of apoptosis *BFAR* and *MDM2* genes were not modulated by the treatments, while *TNFRSF14/HVEM* was significantly downregulated in response to all treatments with respect to control samples (Fig. 6c). Regarding to C5/MJ cells, all the pro-apoptotic genes were slightly or not at all modulated following treatment with AZT alone, but remarkably upregulated, except for *BAX*, following the single Bay 11-7085 or the combined treatments (Fig. 7a). A little differently from what observed for MT-2 cells, in C5/MJ cells all of the anti-apoptotic genes, except for *TNFSF9* and *NOL3*, were downregulated by Bay 11-7085. A specular modulation in C5/MJ cells was exerted on the same anti-apoptotic genes by single treatment with AZT, while all the anti-apoptotic genes were, more or less, upregulated by the combination treatment (Fig. 7a). The bifunctional regulators of apoptosis *BFAR*, *MDM2* and *TNFRSF14/HVEM* were upregulated both by single treatment with AZT and by the combined treatment, when compared to control, but downregulated by single treatment with Bay 11-7085.

**Fig. 8:**
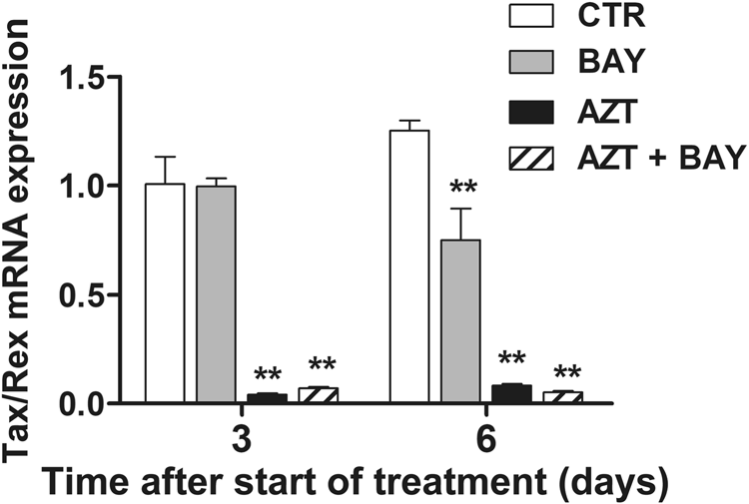
Effects of a combination treatment with AZT and an inhibitor of IκBα phosphorylation on the expression of the HTLV-1 doubly-spliced *Tax/Rex* transcripts in MT-2 cells. MT-2 cells were treated with vehicle (CTR), with 1 µM Bay 11-7085 (BAY), with 128 µM AZT (AZT), or both (AZT+BAY) for a total of 3 days in culture (day 3) or, following a second retreatment with the same protocol, for a total of 6 days in culture (day 6). The histograms represent the mean values ± S.D. from four independent experiments. Asterisks indicate highly significant (**p < 0.001) differences referred to corresponding CTR samples.

The expression of viral genes in HTLV-1-transformed cells in response to the combination treatment with AZT plus the inhibitor of IκBα phosphorylation, was also investigated. Results reported in Fig. 8 refers to the expression of the doubly-spliced *Tax/Rex* transcripts in MT-2 cells at 72 h and 6 days after single or combination treatments, as detected by reverse transcriptase real time PCR (RT-qPCR). Both AZT alone and AZT plus Bay 11-7085, but not Bay 11-7085 alone, remarkably and equally reduced viral gene expression in comparison with control cells as early as at 72 h after treatment. These effects persisted also at 6 days after treatment when a partial, but not significant, reduction in viral transcripts levels was observed even in Bay 11-7085 treated cells.

## Discussion

We have previously demonstrated that knocking out of IκB rendered U937 cells prone to undergo apoptosis when treated with AZT^31^, and that a combination treatment with AZT plus a pharmacological inhibitor of NF-κB induced, in the same cells, apoptotic RCD associated with the suppression of anti-apoptotic genes expression and the upregulation of the mitochondrial apoptotic pathway^32^. Thus, the main achievement of this study was the demonstration that the pharmacological inhibition of NF-κB activation could prompt also HTLV-1 infected/transformed cells towards RCD when treated with a non-toxic dose of AZT. Importantly, results showed that this effect was much less pronounced towards PBMC from healthy donors, and that a similar selective effect could not be obtained by using the compounds of the combination treatment as single agent at whatever concentration.

NF-κB p65/p52 activation, is more heavily regulated in ATL cells than in healthy cells^33^. In addition, the reversible NF-κB inhibitor of p65 translocation, DHMEQ, induced ATL cells to undergo apoptosis in vitro and to inhibit tumor cell growth in vivo in SCID mice^33,34^. Thus, pharmacological inhibition of IκBα phosphorylation has been proposed as a potential strategy to treat and/or prevent ATL^35^. Interestingly, even induction of apoptosis by IFNα/AZT treatment in ATL cells from patients or in ATL cell lines has been associated to suppression of NF-κB activation^20^. Nevertheless, as shown in our dose–effect experiments, highly effective concentrations of pharmacological inhibitors of NF-κB, are not free from toxicity towards normal cells. Moreover, results of EMSA or p65 and p52 binding assays, clearly indicate that single treatment with AZT invariably led to increased levels of NF-κB activation, rather than to a decrease, starting from 24 h after treatment in HTLV-1 transformed cells. This can explain resistance to apoptotic RCD in cells exposed to AZT alone.

To investigate mechanisms underlying the combined effects of AZT plus Bay11-7085, expression of several genes associated to cell death/proliferation were assessed in MT-2 cells, i.e. the HTLV-1-transformed cells that displayed the higher pro-apoptotic response to the combination treatment. Among the pro-apototic genes, of particular interest is the noticeable increase, following the combination treatment, of the expression of the BIM gene, whose product is a good candidate for triggering an intrinsic apoptotic cascade in response to AZT plus Bay 11-7085^36–38^. Moreover, a main difference concerned the expression of anti-apoptotic genes belonging to the *IAP* family, such as *BIRC5/survivin* and *BIRC3/c-IAP2*^39–41^, that were dramatically down modulated in MT-2 cells following the combination treatment. Survivin plays an important role in oncogenesis^42,43^ and a survivin inhibitor, YM155, in combination with anti-CD52 monoclonal antibodies was shown to increase survival in a murine model of human ATLL^44^. Interestingly, a similar, differential effect on anti-apoptotic genes following the combination treatment was not observed in C5MJ. Thus, in general, results of apoptosis-related gene expression indicate that a short-term combination treatment with AZT plus Bay 11-7085 induced the upregulation of pro-apoptotic genes both in MT-2 and in C5/MJ HTLV-1-transformed cells, while preferentially induced the downregulation of most of the anti-apoptotic genes in MT-2 but not in C5/MJ cells. This is in line with a lower pro-apoptotic response to the combined treatment of C5/MJ cells in respect to MT-2 cells, in the short time.

Finally, we cannot help highlighting that the combination treatment with AZT plus Bay 11-7085 significantly reduced levels of pro-viral gene expression in transformed cells. We have not a clear explanation, at the moment, for these data but we can assume that, at the relatively high concentrations utilized, the nucleoside compound could exert also an unexpected inhibitory activity on viral transcripts expression/maturation. The differential expression of several cellular genes, following therapy including RT inhibitors, has been amply documented in HIV-patients^45^. Interestingly, moreover, the capacity of AZT to specifically inhibit retroviral gene expression in cells harboring the HK2 endogenous retrovirus has been reported, suggesting the possibility that the drug could actually affect retroviral mRNA transcription^46^. Our previous study has shown that in ATL patients who responded to therapy with AZT plus IFN, HTLV-1 RT activity was inhibited, while this did not occur in a non-responder patient (ATL)^47^. This implies that AZT actually acts as HTLV-1 RT-inhibitor in vivo. Thus, based on the results we obtained in the present study, we can hypothesize that presence of AZT in a combination therapy for ATL could concomitantly ensure restriction of clonal expansion of the virus and proneness to RCD of HTLV-1 infected cells.

In conclusion, results of the present study represent a proof of concept for the addition of an NF-κB inhibitor to therapeutic regimens including AZT for the treatment of HTLV-1 related diseases.

## Materials and methods

### Cell cultures and reagents

The chronically HTLV-1-infected human cell lines MT-2 (Miyoshi 1981), C91/PL established by co-cultivation of umbilical cord blood T cells with leukemic T cells from an ATLL patient (PL)^48^ and originally obtained from Prof. Robert Gallo (NINCDS NIH Bethesda MD), and C5/MJ (ATCC® CRL-8293™, NIH MD), were maintained in RPMI 1640 medium supplemented with 12% fetal bovine serum (FBS), 50 U/ml streptomycin, 50 U/ml penicillin and 2 mM glutamine (CM; Gibco- Invitrogen, Paisley, Scotland, United Kingdom), in a humidified incubator at 37 °C and 5% CO_2_. The cell lines, here identified as BM24 (CD4+) and BM7 (CD4+/CD8+ double-positive), were previously infected in vitro with HTLV-1 in our laboratory by co-culture of peripheral blood mononuclear cells (PBMC) from healthy donors with irradiated MT-2. BM24 and BM7 were utilized in this study at 12 weeks in culture after infection (a.i), and at 53 weeks in culture a.i.^49^, respectively. BM24 and BM7 were still IL-2 dependent, showing their transformation process was not completed, and were grown in CM in the presence of 20 U of recombinant IL-2 (IL-2; Proleukin, Chiron, Amsterdam, The Netherlands). PBMC were harvested from healthy adult donors seronegative for HIV and hepatitis B and C viruses. Mononuclear cells from heparinized blood were separated using a Ficoll-Hypaque density gradient (Cedarlane, Hornby, Ontario, Canada). PBMC were stimulated with and kept in IL-2 at 20 U/ml for 3 and 6 days, respectively. AZT (Sigma-Aldrich, St. Louis, MO, USA) was diluted in RPMI 1640 and stored at the concentration of 32 mM; Bay 11-7085 (Sigma) was diluted in DMSO and stored at 100 mM. For the combination treatment, cells were usually pre-treated for 2 h with Bay 11-7085 before the addition of AZT. Dosages were chosen on the basis of dose–effect experiments reported in results.

### Apoptosis assay

Apoptosis was assessed by flow cytometry analysis of isolated nuclei following detergent treatment and propidium iodide (PI) staining, using a method that distinguishes nuclei from apoptotic, necrotic or viable cells^50^. Harvested cells were treated with a solution of PI at 25 mg/ml (Sigma-Aldrich) plus 0.05% sodium citrate (Sigma-Aldrich) and with detergent at a high concentration (20% Triton X-100, Sigma-Aldrich) for 30 min and then placed at 4 °C. Isolated nuclei were then analyzed using a FACScan flow cytometry (BD Biosciences), until isolated nuclei were analyzed by fluorescence and by forward- and side-angle-scatter multiparameter analyses using a Becton Dickinson FACS analyzer. Cells for analysis were treated with a mixture consisting of 2% Triton X-100 (Sigma-Aldrich), 25 μg/ml propidium iodide (Sigma-Aldrich) and 0.05% sodium citrate (Sigma-Aldrich). Samples were mixed by gentle inversions for 30 min. A minimum of 5000 events per sample were analyzed. Detectors and amplifier gains for forward and orthogonal scatter were adequately selected to simultaneously detect nuclei from viable, apoptotic and necrotic cells. Events were gated on forward versus orthogonal scatter in such a way that degraded DNA from cell debris or from doublets was excluded and nuclei from viable, apoptotic and necrotic cells were assayed. Data acquisition and analysis were performed using CellQuest software on a minimum of 5000 events for each sample.

### RNA extraction

RNA isolation was performed using a NucleoSpin RNA kit, and to remove possible DNA contamination, RNA was treated with RNase-free DNase according to the manufacturer’s instructions (Machenery-Nagel, Dueren, Germany). The quantity and the quality of all RNA preparations were assessed by gel electrophoresis and optical density at 260 nm/optical density at 280 nm ratios.

### NF-κB-activity assays

For detecting NF-κB activation by non-radioactive electro-mobility shift assay (EMSA), nuclear extracts from cells subjected to different experimental conditions were prepared as follows. Aliquots of 1 × 10^7^cells were washed with cold PBS and suspended in 0.4 ml hypotonic lysis buffer A (10 mM HEPES, pH 7.9, 1.5 mM MgCl_2_, 10 mM KCl, 0.5 mM dithiothreitol, 0.2 mM phenylmethylsulphonyl fluoride, all from Sigma) for 20 min on ice and homogenized by passing through a 25-gauge needle. After centrifugation at 12,000 × *g* for 40 s, nuclear pellets were resuspended in 20 μl ice-cold buffer B (20 mM Hepes, pH 7.9, 25% glycerol, 0.42 M NaCl, 1.5 mM MgCl_2_, 0.2 mM EDTA, 0.5 mM DTT, 0.5 mM PMSF) and supplemented with 1× protease inhibitor cocktail (Roche Applied Science, Indianapolis, IN, USA). Following a 20 min incubation on ice (with recurring mixing), samples were centrifuged at 12,000 × *g* for 5 min and supernatants containing nuclear extracts were collected, aliquoted and immediately stored at −80 °C. Before freezing, protein concentration was assessed by ‘DC Protein Assay’ (Bio-Rad Laboratories, Richmond, CA, USA). The LightShift chemiluminescent EMSA kit (Pierce, Rockford, IL, USA) was utilized to perform the EMSA binding reactions. Specifically, 12 μg of nuclear extracts were incubated with 10 pmoles of double-stranded biotinylated probe containing the NF-κB consensus site, and with 1 μg/μl of poly-dI-dC, to prevent unspecific reaction, in 1× binding buffer for 20 min at room temperature. The complexes were resolved on a 5% native polyacrylamide/0.5× TBE gel and transferred to a positive charge nylon membrane (Bio-Rad Laboratories). After blotting, DNA was cross-linked by UV and signals from the biotin-labeled probe were detected using reagents provided in the ‘LightShift chemiluminescent EMSA module’ (Pierce). Specificity for NF-κB-DNA binding was validated by addition of 100-fold molar excess of unlabeled “cold” specific DNA in reaction mix. Densitometric evaluation of scanned films from three separate experiments by NIH Image J software (version 1.46r, Bethesda, MD, USA) was performed to quantify NF-κB-DNA-binding activity. Data were represented as relative density calculated by the ratio between values obtained for all samples and values of untreated control samples at 24 h.

For quantization of NF-κB p65, p50, and p52 binding by an enzyme-linked immunosorbent assay (ELISA), nuclear extracts from cells subjected to different treatments were obtained as described above. Protein concentration of each sample was determined by BCA protein Assay Kit (Pierce). The DNA-binding activity of NF-κB p65, p50, and p52 was measured using a commercial ELISA according to the manufacturer’s protocol (Trans-AM NF-κB p65 Transcription Factor Assay Kit; Active Motif North America, Carlsbad, CA, USA). The absorbance was determined at 450 nm with wavelength correction 650 nm using a Labsystem Multiskan Bichromatic spectrophotometer (Helsinki, Finland).

### Transcriptional profile by SuperArray

An amount of 3.5 mg of total RNA was used in a reverse transcription (RT) reaction with biotin-16-Dutp (Roche Diagnostics GmbH). RT reaction was performed using Ampolabelling LPR kit (SABioscience Corporation, Frederick, MD, USA). The labeled cDNA was incubated with GEArray-Q Series human apoptosis and cell cycle membranes (SuperArray, SABioscience) at 60 °C overnight. The membrane used in the present study contained 96 genes that were closely related to apoptosis and cell cycle pathways, in addition to positive control genes (glyceraldehydes-3-phosphate dehydrogenase, GAPDH, cyclophillin A, and β-actin). After being washed, the membrane was incubated with streptavidin-alkaline phosphatase and was finally exposed to CDP-Star chemiluminescent substrate (SuperArray). Signal detection was performed using a high Performance chemiluminescence film (Amersham Biosciences). Analysis of results was performed by GEArray Expression Analysis Suite software (http://geasuite.superarray.com). According to this analysis, transcriptional levels of genes showing fold change values of >1.50 or <0.66 were considered significantly modified.

### Real-time quantitative reverse transcription PCR

The transcriptional activity of the genes of interest was evaluated by real-time quantitative reverse transcription PCR (RQ-PCR), using a CFX-96 real-time instrument (Bio-Rad) and cDNA specific primers purchased from Primm (Milan, Italy). The list of genes assayed and the corresponding sequences of the primers utilized are reported in Supplementary Information (SI) 1. To evaluate HTLV-1 mRNA expression, RPXPR1 and RPX4 primers specific for the Tax/Rex splicing region were used^51^. For reverse-transcription, 0.25 μg of total RNA extracted from each experimental sample were processed for cDNA generation using a high capacity cDNA Reverse Transcription Kit (Applied Biosystems), according to the manufacturer’s instructions. RQ-PCR was performed as previously described in detail^52^ and mRNA levels were calculated referring to glucuronidase beta (GUSB) as a housekeeping gene. Then, to compare the expression of each gene in the different treatment conditions, the relative expression was calculated using the equation 2^−ΔΔCt^ where ΔΔCt = ΔCt (treated) − ΔCt (control), and ∆Ct (treated) = [Ct (target gene) − Ct (GUSB)]treated as well as ∆Ct (control) = [Ct (target gene) − Ct (GUSB)]control.

### Statistical analysis

Each experiment was performed in triplicate with different replicates for sample, except one case when only two independent experiments were performed, as stated in the related legend of the Fig. 8. All data are presented as mean ± S.D. Data analysis was performed using the SPSS statistical software system (version 17.0 for Windows, Chicago, IL). For comparison of the means, the Bonferroni’s post-hoc multiple comparison ANOVA test was utilized. Results of the statistical tests are reported in the figures as: (*) for *p* < 0.05 and (**) for *p* < 0.001.

## Supplementary information

Primer pairs used in quantitative reverse transcription-polymerase reaction analysis.

Flow cytometry analysis following staining with Annexin-V/PI and Western blot analysis of caspase-3 and PARP-1 cleavage of samples from MT-2 cells subjected to combination treatment.

Detection of NF-κB activation in C5/MJ cells treated with AZT and an inhibitor of IκBα phosphorylation.

mRNA changes determined by SuperArray in MT-2 cells exposed for 24h to a combination treatment with AZT plus Bay 11-7085
