# Supplementary material for: Inhibition of IκBα phosphorylation potentiates regulated cell death induced by azidothymidine in HTLV-1 infected cells
Source: Cell Death Discov. 2020 Feb 18;6:9. doi: 10.1038/s41420-020-0243-x (PMC7028944; doi:10.1038/s41420-020-0243-x)
Supplement: Supplementary file 1 — Primer pairs used in quantitative reverse transcription-polymerase reaction analysis. [file 41420_2020_243_MOESM1_ESM.pdf]

SUPPLEMENTARY INFORMATION 1

SI 1. Primer pairs used in quantitative reverse transcription-polymerase reaction analysis.

**GenBank accession number**	**Gene transcripts**	**Gene functional family**	**Primers sequence 5'-> 3'**
NM_001160	*APAF1*	CARD family	Fw: ATCCTGGTGCTTTGCCTCTA<br>Rv: TACACCCCCTGAAAAGCAAC
NM_004324	*BAX*	Bcl-2 protein family	Fw: CCAAGGTGCCGGAACTGA<br>Rv: CCCGGAGGAAGTCCAATGT
NM_000633	*BCL2*	Bcl-2 protein family	Fw: TCCCTCGCTGCACAAATACTC<br>Rv: ACGACCCGATGGCCATAGA
NM_006538	*BCL2L11/BIM*	Bcl-2 protein family	Fw: AGAGCCACAAGACAGGAGCCCA<br>Rv: CAGCCTGCCTCATGGAAGCCA
NM_004050	*BCL2L2/BCL-W*	Bcl-2 protein family	Fw: CCTAGAATGTGGCAACGTAGTTGT<br>Rv: TCAACACCTGAGGCCAATTTG
NM_016561	*BFAR*	Death effector domain family	Fw: TGGTCAAATACTCCTTCCTTCCAT<br>Rv: CCAACCAGTCCCAAGCAAA
NM_001166	*BIRC2/c-IAP1*	IAP family	Fw: CCTGTGGTGGGAAGCTCAGT<br>Rv: CCTCCGGTGTTCTGACATAGC
NM_001165	*BIRC3/c-IAP2*	IAP family	Fw: CAAAGCATTGAAGTCTGCAGTTG<br>Rv: GCAAGCATGGTTTCCTGGAT
NM_001168	*BIRC5/survivin*	IAP family	Fw: TGCTGCAGGCCGTGTGT<br>Rv: TCTCCCCCGTGTGGAGAAC
NM_001137	*CASP8AP2/FLASH*	CASPASE family	Fw: CACTTGCCACTTCTACAAGTC<br>Rv: TGGCGGCTAAATATGCAAATG
NM_001250	*CD40*	TNF receptor family	Fw: TGTCCTGCACCGCTCATG<br>Rv: ACCCCTGTAGCAATCTGCTTGA
NM_001279	*CIDEA*	CIDE and death domain	Fw: CAGGCTGAACCCCAAGGA<br>Rv: TCTCATACATGGTGGCCTTCAC
NM_014430	*CIDEB*	CIDE and death domain	Fw: AGCCAAAGCATTGGAGACCCTACT<br>Rv: TCTGACCAGACTGCAACACCATCA
NM_003824	*FADD*	Death effector domain family	Fw: GCTGGCTCGTCAGCTCAAA<br>Rv: ACTGTTGCGTTCTCCTTCTCT
NM_000181	*GUSB*	Housekeeping gene	Fw: CAGTTCCCTCCAGCTTCAATG<br>Rv: ACCCAGCCGACAAAATGC
NM_002392	*MDM2*	DNA damage family	Fw: GACTCCAAGCGCGAAAACC<br>Rv: ACATGTTGGTATTGCACATTTGC
NM_003946	*NOL3*	CARD family	Fw: AGTTCGAAGAAATGGGCAAC<br>Rv: GCATCCAAGGCTTCGTACTC
NM_001561	*TNFRSF9/4-IBB-L*	TNF receptor family	Fw: TTGGGAACATTTAATGACCAGA<br>Rv: TCCCGGTCTTAAGCACAGAC
NM_003842	*TNFRSF10B/DR5*	TNF receptor family	Fw: GGGCCACAGGGACACCTT<br>Rv: TCGCCCGGTTTTGTTGA
NM_003807	*TNFRSF14/HVEM*	TNF ligand family	Fw: GCTGGATGAACGCCTGGTT<br>Rv: CATGAAAGCCCCGAAGTAAGAC
NM_001167	*XIAP/BIRC4*	IAP family	Fw: TTTTTCCCCTGTCCCTTTGA<br>Rv:ACACAGGGCCAAATCACATTATATAC
