# Supplementary material for: Inhibition of IκBα phosphorylation potentiates regulated cell death induced by azidothymidine in HTLV-1 infected cells
Source: Cell Death Discov. 2020 Feb 18;6:9. doi: 10.1038/s41420-020-0243-x (PMC7028944; doi:10.1038/s41420-020-0243-x)
Supplement: Supplementary file 2 — Flow cytometry analysis following staining with Annexin-V/PI and Western blot analysis of caspase-3 and PARP-1 cleavage of samples from MT-2 cells subjected to combination treatment. [file 41420_2020_243_MOESM2_ESM.pdf]

SUPPLEMENTARY INFORMATION 2

SI 2. Flow cytometry analysis following staining with Annexin-V/PI and Western blot analysis of caspase-3 and PARP-1 cleavage of samples from MT-2 cells subjected to combination treatment with AZT and an inhibitor of IκBα phosphorylation (Bay 11-7085).

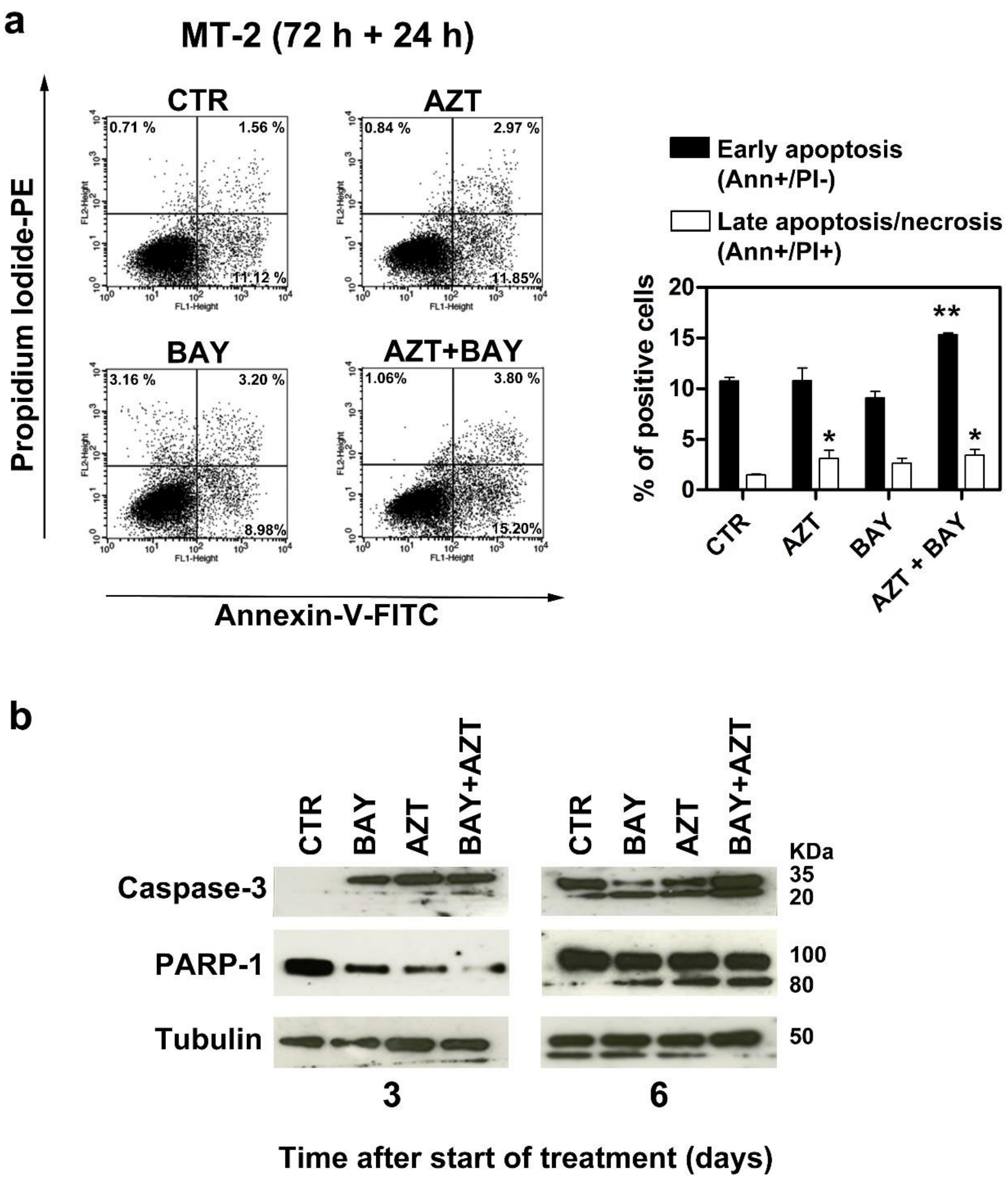

SI 2. (a) MT-2 cells were treated with vehicle (CTR), with 1 µM Bay 11-7085 (BAY), with 128 µM AZT (AZT), or both (AZT+BAY) for 3 days in culture and, following a second retreatment with the same protocol, for a further 24 h. Apoptosis was then quantified by two-fluorescence flow cytometry analysis of the cells following staining with Annexin-V-FITC/propidium iodide (PI), using a FACScan flow cytometry (BD Biosciences)**.** Note that, in these experiments, time in culture after the last treatment was not prolonged to day 3, as done for experiments reported in (b) of this Supplementary Information or in Figure 3 of the main text, in order to limit as much as possible the occurrence of late apoptosis that is not distinguishable from necrosis using the Annexin-V/PI technique. Obviously, the different kinetic also accounts for lower levels of apoptosis with respect to those reported in Figure 3 of the main text. Representative flow cytometric cytograms and histograms that represent the mean values ± S.D, from three independent assays, of Annexin-V+/PI+ cells (late apoptosis/necrosis) and Annexin-V+/PI- cells (early apoptosis). Asterisks indicate highly significant (**$p < 0.001$) or significant (*$p < 0.05$) differences referred to corresponding CTR samples. **(b)** MT-2 cells were treated with vehicle (CTR), with 1 µM Bay 11-7085 (BAY), with 128 µM AZT (AZT), or both (AZT+BAY) for a total of 3 days in culture (day 3) or, following a second retreatment with the same protocol, for a total of 6 days in culture (day 6). Caspase-3 and PARP-1 cleavage was detected in protein extracts from the same cells by Western blot analysis. For protein extraction, MT-2 cells were collected and lysed. Proteins extracted were then analyzed through SDS–PAGE and probed with rabbit polyclonal antibody against human caspase-3 (1:3000, BD Bioscience Pharmingen), rabbit polyclonal antibody against PARP-1 (cleaved Asp214, Asp215) (1:2000, Invitrogen), and goat polyclonal antibody human against Tubulin (1: 3000 Novus Biologicals, Inc Littleton, CO) followed by detection with ECL plus (GE Healthcare) as previously described [Cordero FM, Bonanno P, Khairnar BB, Cardona F, Brandi A, Macchi B, Minutolo A, Grelli S, Mastino A. (-)-(1R,2R,7S,8aR)-1,2,7-Trihydroxyindolizidine ((-)-7S-OHLentiginosine): synthesis and proapoptotic activity. ChemPlusChem. 2012, 77: 224-233]. Digital images were obtained using the ChemiDoc XRS+ and Image Lab software (Bio-Rad, CA, USA).
