# Supplementary material for: Inhibition of IκBα phosphorylation potentiates regulated cell death induced by azidothymidine in HTLV-1 infected cells
Source: Cell Death Discov. 2020 Feb 18;6:9. doi: 10.1038/s41420-020-0243-x (PMC7028944; doi:10.1038/s41420-020-0243-x)
Supplement: Supplementary file 3 — Detection of NF-κB activation in C5/MJ cells treated with AZT and an inhibitor of IκBα phosphorylation. [file 41420_2020_243_MOESM3_ESM.pdf]

SUPPLEMENTARY INFORMATION 3

SI 3. Detection of NF-κB activation in C5/MJ cells treated with AZT and an inhibitor of IκBα phosphorylation.

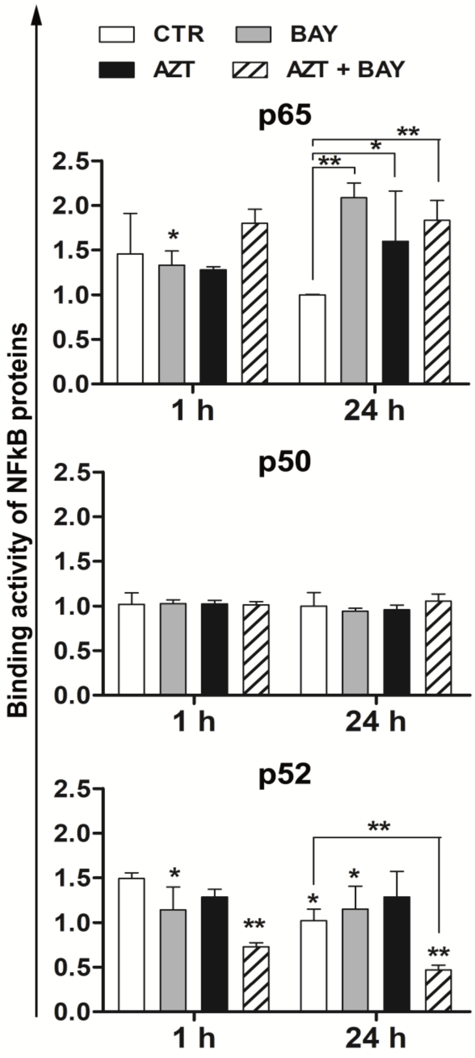

SI 3. C5/MJ cells were either treated with vehicle (CTR) or treated with 1 µM Bay 11-7085 alone (BAY), 128 µM AZT alone (AZT), or with both (AZT+BAY), and then assayed at 1 h and 24 h after the last treatment for detection of phosphorylated p65, p50 and p52 DNA binding by an enzyme-linked immunosorbent assay (ELISA) The histograms represent the mean values ± S.D. from three independent experiments and are expressed as the ratio of the values obtained from samples of the experimental groups versus those obtained in samples of the CTR group at 24 h. Asterisks indicate significant (*$p < 0.05$) and highly significant (**$p < 0.001$) differences referred to the CTR group at 1 h (no connection bar) or between groups (connection bars).
