# Supplementary material for: Inhibition of IκBα phosphorylation potentiates regulated cell death induced by azidothymidine in HTLV-1 infected cells
Source: Cell Death Discov. 2020 Feb 18;6:9. doi: 10.1038/s41420-020-0243-x (PMC7028944; doi:10.1038/s41420-020-0243-x)
Supplement: Supplementary file 4 — mRNA changes determined by SuperArray in MT-2 cells exposed for 24h to a combination treatment with AZT plus Bay 11-7085 [file 41420_2020_243_MOESM4_ESM.pdf]

SUPPLEMENTARY INFORMATION 4

SI 4. mRNA changes determined by SuperArray in MT-2 cells exposed for 24h to a combination treatment with AZT 128 μM plus Bay 11-7085 1 μM.

Gene transcripts	GenBank accession number of transcript sequence	Expression Levels Fold change[1] (AZT + BAY/CTR)	Gene functional family
**Pro-apoptotic**			
**UP**			
TNRSF10A/DR4	NM_003844	3.23	TNF receptor family
TRAIP	NM_005879	1.72	TRAF family
TRAF6	NM_004620	1.56	TRAF family
CASP2	NM_032982	7.03	Caspase family
CASP5	NM_004347	2.52	Caspase family
CASP6	NM_032992	2.15	Caspase family
TNFRSF25/DR3	NM_003790	17.21	CARD family
APAF1	NM_001160	3.32	CARD family
CRADD	NM_003805	2.15	CARD family
FADD	NM_003824	3.89	Dead Effector Domain family
CIDEA	NM_001279	3.32	CIDE domain family
CIDEB	NM_014430	4.68	CIDE domain family
BCL2L11/BIM	NM_006538	2.2	Bcl-2 family
**DOWN**			
TNFRSF1A	NM_001065	0.33	TNF receptor family
TNFRSF10B/DR5	NM_003842	0.51	TNF receptor family
CD40	NM_001250	0.37	TNF receptor family
TNFRSF9/4-1BB	NM_001561	0.6	TNF receptor family
BAX	NM_004324	0.62	Bcl-2 family
CASP7	NM_001227	0.3	Caspase family
CASP8AP2 /FLASH	NM_012115	0.45	Dead Effector Domain family
DFFA	NM_004401	0.42	CIDE domain family
DFFB	NM_004402	0.74	CIDE domain family
**Anti-apoptotic**			
**UP**			
BNIP3	NM_004052	29.05	Bcl-2 family
TNFRSF10D/DcR2	NM_003840	1.93	TNF receptor family
BCL2	NM_000633	2.24	Bcl-2 family
BCL2A1/BFL-1	NM_004049	6.87	Bcl-2 family
HRK	NM_003806	1.67	Bcl-2 family
TNFSF9/4-1BB-L	NM_003811	1.72	TNF ligand family
**DOWN**			
NOL3	NM_003946	0.34	CARD family
NAIP/BIRC1	NM_004536	0.18	IAP family
BIRC3/c-IAP2	NM_001165	0.27	IAP family
BIRC6/BRUCE	NM_016252	0.3	IAP family
BIRC2/c-IAP1	NM_001166	0.31	IAP family
BIRC5/survivin	NM_001168	0.66	IAP family
XIAP/BIRC4	NM_001167	0.47	IAP family
BCL2L2/BCl-W	NM_004050	0.28	Bcl-2 family

Multifunctional

**UP**			
TNF	NM_000594	4	TNF ligand family
TRAF2	NM_021138	2.78	TRAF family
BFAR	NM_016561	45.77	Dead Effector Domain family
**DOWN**			
TNFRSF14/HVEM	NM_003807	0.4	TNF ligand family
MDM2	NM_002392	0.33	ATM and p53 family

[1] Data derive from results of three independent experiments
